# Removal of Hexavalent Chromium from Wastewater Originating from Spent Bricks by Modified Biochars Derived from Honeybee Biomass

**DOI:** 10.3390/molecules30112421

**Published:** 2025-05-31

**Authors:** Rafał Olchowski, Kinga Morlo, Joanna Dobrzyńska, Ryszard Dobrowolski

**Affiliations:** 1 Department of Pharmacology, Toxicology and Environmental Protection, Faculty of Veterinary Medicine, University of Life Sciences, Akademicka St. 12, 20-950 Lublin, Poland; rafal.olchowski@up.lublin.pl; 2 Department of Analytical Chemistry, Institute of Chemical Sciences, Faculty of Chemistry, Maria Curie-Sklodowska University, M. C. Sklodowska Sq. 3, 20-031 Lublin, Poland; kinga.morlo@mail.umcs.pl (K.M.); joanna.dobrzynska@mail.umcs.pl (J.D.)

**Keywords:** beekeeping waste, carbonaceous material, pyrolysis, hexavalent chromium, adsorption mechanism

## Abstract

The removal of Cr(VI) from wastewater is a crucial task due to its high toxicity. In this study, slumgum-originated biochar materials were obtained by three different methods: high-temperature pyrolysis with H_3_PO_4_ or CO_2_ and the high-temperature treatment of CO_2_-activated slumgum-originated biochar in an Ar atmosphere. The obtained materials were subjected to physicochemical characterization (nitrogen adsorption/desorption isotherms, CHN elemental analysis, Fourier transform infrared spectroscopy, and X-ray photoelectron spectroscopy) and tested for their adsorption properties towards Cr(VI) ions. The solution pH, contact time, and effects of the Cr(VI) concentration on Cr(VI) adsorption onto biochar materials were studied. The kinetics and isotherm experimental data were best fitted to the Elovich (R^2^ = 0.848) and Freundlich (R^2^ = 0.965) theoretical models for H_3_PO_4_-modified biochar. The highest adsorption capacity (45.0 mg g^−1^) for Cr(VI) was obtained for biochar modified with H_3_PO_4_. It was stated that the relatively fast rate of Cr(VI) adsorption onto this biochar (equilibrium reached within 120 min) is related to its mesoporous structure. The mechanism of Cr(VI) adsorption onto H_3_PO_4_-modified biochar was studied in detail. The obtained biochar was successfully applied for efficient Cr(VI) removal from wastewater originating from spent bricks with a low biochar dosage (4.0 g L^−1^).

## 1. Introduction

Slumgum is a kind of organic agro-industrial waste derived from beekeeping farms. The waste is made up of used honeybee frames. This dark brown material may contain brood cocoons, moths, and larvae of *Galleria mellonella* L., dead bees, excrement, pollen, propolis, and non-extractable beeswax. Additionally, pesticides and heavy metals (such as Fe, Cr, and Ni) can occur in the slumgum. Pesticides (e.g., *Coumaphos*) are carried by bees along with pollen from farmlands, where agricultural chemicals are broadly used, and can be applied by beekeepers inside the hives (amitraz) against a parasitic disease known as varroosis, which is caused by *Varroa destructor*. A source of heavy metals is beekeeping wires. The worldwide production of slumgum is estimated to be 55.5 thousand tons annually [1,2,3]. Currently, slumgum is not recycled. The improper management of this waste can lead to soil and groundwater contamination by toxic substances (pesticides, heavy metals) and biohazard residues (fungi, insects, bacteria, etc.), creating a serious problem. Thus, the beneficial use of slumgum can solve this problem. There are some studies regarding the application of slumgum for plant production as a compost [2] or for energy production (estimated heating value: 17.076 MJ kg^−1^ in the dry base) [4]. Another possibility is using this waste, after its conversion into biochar, for pollutant removal (e.g., Cr(VI)) from the environment.

Biochar is a low-cost carbon-rich adsorbent obtained through the pyrolysis of organic waste at a temperature above 250 °C in anaerobic or almost anaerobic conditions. Generally, pristine biochar is a slightly porous solid; thus, its appropriate modification is necessary to obtain materials (engineered biochar) that possess a high surface area and some active surface functional groups [5]. In the literature, many papers consider using biochar for Cr(VI) removal from aqueous solutions. Various types of biochar have been used to remove Cr(VI) from water: Fe-loaded biochar derived from waste zanthoxylum branch [6], rice husk biochar modified with MgO [7], walnut shell biochar modified with β-cyclodextrin and chitosan, bagasse powder biochar modified with Zn(NO_3_)_2_, wheat straw biochar modified with FeS and carboxymethylcellulose [8], wetland reed or herb residue biochar modified with nanoscale zero-valent iron [9], carbonized hemp seeds impregnated with Fe_3_O_4_ nanoparticles [10], bamboo biochar, water hyacinth biochar [11], *Melia azedarach* wood derived biochar [12], *Pinus radiata* forest residue biochar [13], and tobacco petiole pyrolytic biochar [14]. Beekeeping waste has not yet been used for biochar synthesis. The slumgum can be turned into biochar because its carbon content is nearly 50% wt. Furthermore, biochar obtained from slumgum waste can contain some heteroatoms, such as O and N, in its structure, which can act as surface active sites for the adsorption of heavy metal ions [2,3]. Thus, it is an ideal candidate for efficiently removing heavy metal ions, such as Cr(VI) anions, from wastewater.

Cr(VI) ions are the most toxic chromium species due to their permeability through biological membranes and their strong oxidative properties. Cr(VI) ions are extensively introduced into the environment, along with wastewater, from mining, leather tanning, electroplating, steel and rubber manufacturing, and the textile industry. Another anthropogenic source of Cr(VI) is spent magnesia–chrome bricks. Magnesia–chrome bricks are widely used in many industries due to their attractive mechanical and refractory properties. However, owing to their chemical corrosion, thermal shock, and mechanical damage, the used bricks generally have to be replaced every several months to two years, depending on their application. Millions of tons of spent MgO-Cr_2_O_3_ bricks are generated annually worldwide. It is worth mentioning that about 90% of them are landfilled with or without treatment. Chromium from the spent bricks can be transferred into soil and water, mainly as Cr(VI) species. The Cr(VI) concentration in the water originating from these bricks can significantly exceed the World Health Organization’s maximum allowed concentration of total chromium in drinking water, i.e., 50 µg L^−1^. Prolonged contact with the Cr(VI) can lead to allergic reactions, kidney and liver damage, skin rashes, genetic alterations, various kinds of cancers, and death [15,16,17,18,19,20,21]. In south-eastern Poland, a huge cement plant generated thousands of tons of used chromite–magnesite bricks without recycling them for several decades. Soil and groundwater have been contaminated over a large area. An effective method for the remediation and purification of local drinking water is being sought [22].

In this work, three biochars were synthesized from bee waste. The first one was synthesized from wax-free slumgum in a CO_2_ atmosphere; for the synthesis conditions, including the use of CO_2_, we aimed to obtain a material rich in mesopores [23]. Slumgum was impregnated with phosphoric acid to prepare the second carbon and, after drying, pyrolyzed in a nitrogen atmosphere [24]. The third material was obtained due to the thermal reduction of the first material (obtained in CO_2_) in an argon atmosphere. The obtained biochar materials were subjected to physicochemical characterization and compared in terms of their adsorption properties to Cr(VI) ions. The experiments, which evaluated the pH effect, effect of contact time on Cr(VI) adsorption, and determination of adsorption isotherms, were carried out. The biochar with the best Cr(VI) removal characteristics was used for the decontamination of wastewater originating from spent bricks. The adsorption mechanism for the material with the best adsorption properties towards Cr(VI) was examined.

## 2. Results and Discussion

### 2.1. Physicochemical Studies

**Figure 1:**
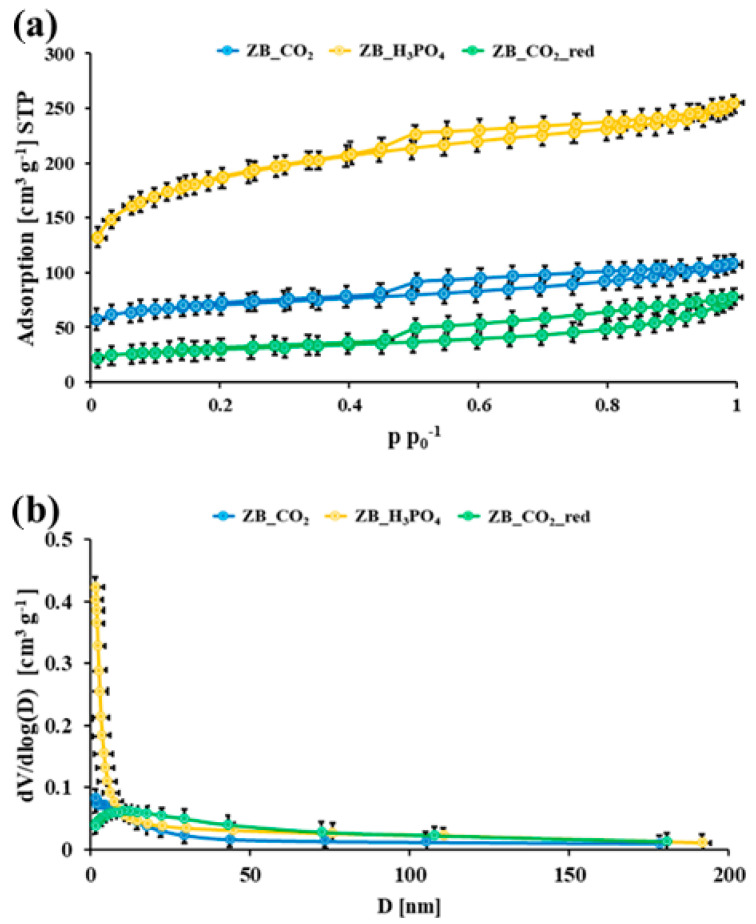
(**a**) Nitrogen adsorption/desorption isotherms and (**b**) BJH pore size distributions for the studied materials; error bars denote standard deviations from three replicates.

In Figure 1a and Figure S1, the nitrogen adsorption/desorption isotherms for the pristine slumgum material and the studied biochar materials are presented, respectively. The pristine slumgum material was practically nonporous. On the contrary, the synthesized biochar samples were characterized by the IVa-type nitrogen adsorption/desorption isotherms, with the hysteresis loop of H1 type at about 0.45 p p_0_^−1^, according to the IUPAC [25]. The shape of the nitrogen adsorption/desorption isotherms and the hysteresis loop for each biochar suggested that the synthesized materials had a micro-mesoporous structure.

Table 1 shows the physicochemical parameters of the pristine material and the studied biochar materials. These data confirm the nonporous nature of the pristine slumgum material (ZB). Conversely, the activation/modification of the pristine slumgum resulted in the porous biochar samples. The high S_BET_ values for all obtained biochar materials suggest their developed microporous structure. The highest specific surface area was obtained for the ZB_H_3_PO_4_ material (639 m^2^ g^−1^), and the lowest was obtained for the ZB_CO_2__red material (105 m^2^ g^−1^). The same tendency could be observed for the total pore volume values (0.39 cm^3^ g^−1^ for ZB_H_3_PO_4_ and 0.12 cm^3^ g^−1^ for ZB_CO_2__red). Considering the high value of the specific surface area and the smallest average pore volume of the ZB_H_3_PO_4_ material, it can be concluded that it has the most significant micropores. In Figure 1b and Figure S2, the pore size distributions (PSDs) for all studied materials are presented. The course of the curve showing the pore size distribution also confirms the presence of a significant number of micropores in the ZB_H_3_PO_4_ material. The other two materials also contain micropores, but there are fewer of them than in ZB_H_3_PO_4_. Comparing the ZB_CO_2_ and ZB_CO_2__red materials, it can be concluded that the former has a more developed surface and more micropores than the latter. The ZB_CO_2__red material, characterized by the largest pore diameter (5.7 nm), contains more mesopores than ZB_CO_2_. The following reduction of the ZB_CO_2_ biochar under an Ar atmosphere at 1000 °C caused the micropore population to decrease. Furthermore, comparing the ZB_CO_2_ and ZB_H_3_PO_4_ materials, it can be concluded that the H_3_PO_4_ activation method much more readily allowed for the development of the micropore structure than CO_2_ activation.

According to the porosity data, the most efficient method for activating/modifying the pristine slumgum was the one based on the use of the H_3_PO_4_ porosity agent, which is broadly described in the literature [26,27,28,29,30,31]. The H_3_PO_4_ initiated a variety of reactions within the carbon precursor (e.g., dehydration, elimination). Some volatile species, such as CO_2_ and H_2_O, were produced during these processes. They penetrated the structure of the pristine material and formed pores. Moreover, the P-species could be simultaneously bonded to the surface of the forming biochar and constitute the binding active sites [29]. The production of volatile species inside the structure of slumgum during H_3_PO_4_ treatment was a more efficient way of activating it than the hindered diffusion of CO_2_ from the gas phase throughout the packed deposit. The slumgum material previously physically activated with CO_2_ also lost its microporosity after high-temperature treatment under an Ar atmosphere. This is probably related to the micropore blockage caused by the solid pyrolysis products formed in these conditions.

According to the elemental analysis data (CHN and XPS, Table 2), the pristine slumgum material contains 59.2 wt. % of carbon, 10.7 wt. % of hydrogen and 5.2 wt. % of nitrogen. The various substances present in slumgum (e.g., waxes, honey, pesticides) could be the primary sources of these elements. The CO_2_ activation of the pristine slumgum resulted in a nitrogen content decrease (to 3.8 wt. %). Some of the nitrogen-containing groups could be removed from this material at high temperatures. The ZB_CO_2_ material possessed a relatively low oxygen content (7.6 wt. %) and some other elements: P (1.9 wt. %) and K (4.9 wt. %), which could be related to the pristine slumgum material. The high-temperature reduction of ZB_CO_2_ material in the argon atmosphere increased the oxygen content from 7.6 wt. % to 17.2 wt. % and decreased the carbon (from 84.7–88.8 wt. % to 68.3–71.4 wt. %) and hydrogen (from 2.9 wt. % to 1.6 wt. %) content. The above changes in the content of carbon, oxygen and hydrogen, with simultaneous relatively small changes in the content of mineral components such as phosphorus and potassium, indicate that as a result of heating ZB_CO_2_ in an argon atmosphere, some carbon (especially from the near-surface layers) passes into a gaseous state and is removed from the structure. The increase in the oxygen content in the ZB_CO_2__red material determined by the XPS technique indicates that oxides, mainly potassium oxide, may be present on the surface. The content of potassium in ZB_CO_2__red, determined by the XPS technique, is 5.1%. In the case of both ZB_CO_2_ and ZB_CO_2__red, the difference in the nitrogen content obtained by various methods (CHN (3.8–3.9 wt. %) and XPS (2.1–2.6 wt. %)) could be due to its presence on the inner structure of the material rather than on its surface. Activation using phosphoric acid increased the phosphorus content to 8.1% in ZB_H_3_PO_4_, which was about 4 times higher than in CO_2_-activated carbons. A significant difference between acid and CO_2_-activated carbon is the almost complete decrease in the potassium content in ZB_H_3_PO_4_, which can be presumed to have been converted into a readily soluble form and removed from the structure during the washing of the pyrolysis product. FT-IR and XPS studies deliver more detailed information about the surface functional groups present in the synthesized biochar materials. Figure S3 presents the FT–IR spectra of the pristine ZB and synthesized biochar materials. There were some spectral bands located at 3437 cm^−1^, 2954–2852 cm^−1^, 1738 cm^−1^, 1651–1639 cm^−1^, 1550–1541 cm^−1^, 1463–1461 cm^−1^, 1384–1376 cm^−1^, 1262–1031 cm^−1^ and 720–669 cm^−1^, which corresponded to ν_OH,NH_, ν_CH_, ν_C=O_ (ester bond), ν_C=O,C=C_ (aromatic ring), δ_NH_, δ_CH_, δ_OH_, ν_C–O,C–N, P–O_ and γ_Ar,ArH_ [32,33,34,35,36,37]. During the physical activation of ZB by carbon dioxide (Figure S3a), the content of hydroxyl groups increased, which could be observed by the higher intensity of corresponding spectral bands. Simultaneously, carbonyl, carboxyl, amine groups, and the aliphatic carbon content decreased. The observed changes in the FT–IR spectra could be related to the oxidation of aliphatic carbon chains, the partial removal of amine groups, and the breakup of C=O groups by creating new C–O groups. Moreover, the decrease in the intensity of C=C aromatic bands suggested that graphene domains were formed during the CO_2_ modification of the ZB material. In the graphene domain, the C=C vibrations could be substantially hindered. Similar observations were made in the case of the H_3_PO_4_ modification of the raw ZB material. Additionally, the band’s intensity at 1262–1031 cm^−1^ drastically increased, which could be related to incorporating additional P–O functional groups into the surface of the modified biochar. In turn, the high-temperature treatment of ZB_CO_2_ biochar under an argon atmosphere decreased the content of all surface functionalities, probably due to the thermal desorption process (Figure S3b).

**Figure 2:**
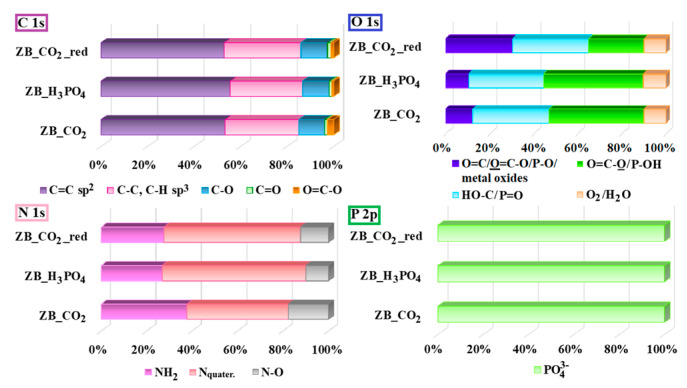
The share of individual forms of C, O, N, and P determined based on the XPS data for the studied biochar materials.

High-resolution XPS spectra for the studied biochar samples were recorded in the case of the C 1s, O 1s, N 1s, and P 2p core energy level bands. The deconvolution of the C 1s core energy level band revealed five signals located at 284.5 eV (C=C sp^2^, aromatic carbon), 285.3 eV (C–C, C–H sp^3^, aliphatic carbon), 286.6 eV (C–O), 287.9 eV (C=O, carbonyl), and 289.7 eV (O=C–O, carboxyl) [38,39]. For the O 1s core energy level, four signals were obtained, which were located at 531.1 eV (O=C/O=C–O/O–P), 532.6 eV (HO–C/O=P), 533.6 eV (O=C–O, P–OH), and 535.4 eV (adsorbed O_2_/H_2_O) [40,41,42,43]. For the N 1s core energy level, three signals were obtained, which were located at 398.9 eV (NH_2_, amine/pyridine), 400.9 eV (N_quater._, quaternary nitrogen), and 403.0 eV (N–O, nitrogen oxide/nitro) [44,45,46]. Finally, one doublet signal was obtained for the P 2p core energy level (P 2p_3/2_ and P 2p_1/2_). The P 2p_3/2_ signal was located at 133.8 eV (PO_4_^3−^, phosphates) [47]. The intensity contributions of each deconvoluted signal in the C 1s, O 1s, N 1s, and P 2p XPS core energy levels for the studied biochar materials are presented in Figure 2. For all materials, the main intensity contribution in the C 1s band (53.0–55.6%) was related to aromatic C=C sp^2^ from the graphene domains. In the case of H_3_PO_4_ treatment for the ZB material, the C=C sp^2^ content was the highest (55.6%), which suggests the most efficient carbonization method. Additionally, an increase in the intensity contribution for C–C, C–H sp^3^ (from 31.7% to 33.0%) and a decrease in the intensity contribution for O=C–O (from 2.6% to 1.1%) were observed during the high-temperature treatment of ZB_CO_2_ biochar. This could be related to the thermal desorption of COOH groups from the carbonaceous surface. The relatively high-intensity contribution of C–C, C–H sp^3^ (31.0%) was also observed for ZB_H_3_PO_4_ biochar. The intensity contribution of other C 1s signals was similar for all studied materials (11.1–11.5% for C–O and 1.2–1.5% for C=O). According to the O 1s band data for ZB_CO_2_ and ZB_CO_2__red biochar materials, it could be observed that the high-temperature treatment of ZB_CO_2_ biochar resulted in a significant increase in the content of the O=C/O=C–O/metal oxides groups (from 11.8% to 29.7%) and a slight decrease in the content of HO–C/P=O (from 35.3% to 33.4%) and O=C–O/P–OH (from 44.3% to 24.6%). A different situation was observed for ZB_H_3_PO_4_ biochar. The highest intensity contribution of the O 1s band was observed for O=C–O/P–OH (45.3%), which could be related to incorporating phosphoric acid groups into the surface of the studied biochar. In all cases, the highest intensity contribution in the N 1s band was registered for N_quater._, especially for ZB_H_3_PO_4_ and ZB_CO_2__red (60.1–63.2%). It is likely that the thermo-desorption of NH_2_ and N–O groups and the incorporation of N atoms into the graphene layers could take place during the H_3_PO_4_ treatment of ZB material and the high-temperature heating of ZB_CO_2_ biochar in an inert atmosphere. Finally, P was present as the PO_4_^3−^ for all studied materials.

To examine the acid–base properties of the studied biochar materials, the pH_pzc_ of the biochar was determined. The following pH_pzc_ values were obtained: 9.5 (ZB_CO_2_), 10.7 (ZB_CO_2__red), and 4.1 (ZB_H_3_PO_4_). Thus, the surface of ZB_CO_2_ and ZB_CO_2__red possessed a basic nature. The ZB treatment with H_3_PO_4_ resulted in the most acidic biochar, which could be related to the incorporation of many phosphorus groups on the surface of the synthesized biochar and the presence of COOH surface groups. Biochar activated by CO_2_ had a less acidic surface, without as many phosphorus groups as ZB_H_3_PO_4_. Moreover, the high-temperature treatment of ZB_CO_2_ biochar in an Ar atmosphere resulted in the basic character of the ZB_CO_2__red surface. This could have resulted from the removal of carbon-containing components and the increase in the surface content of basic oxides, e.g., potassium oxide, which is evidenced by the rise in the oxygen signal intensity in the XPS spectra of ZB_CO_2__red.

### 2.2. Cr(VI) Adsorption Tests

**Figure 3:**
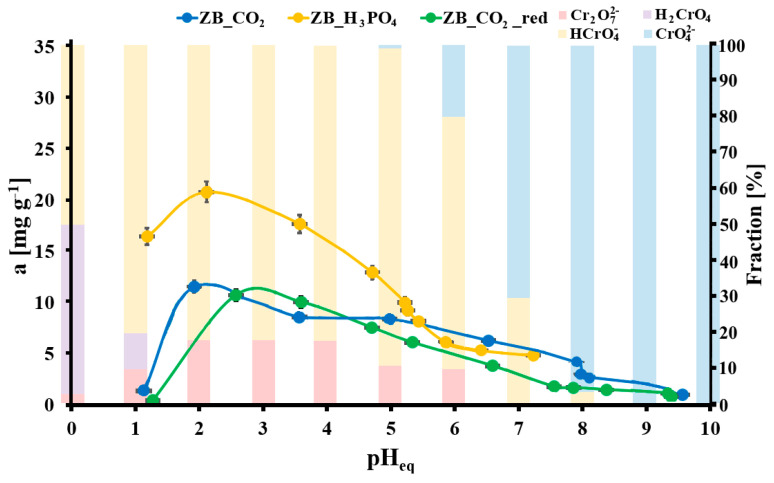
Cr(VI) adsorption onto studied biochar materials vs. pH of the aqueous solution (m = 20 mg, V = 5 mL, C_inCr(VI)_ = 100 mg L^−1^, t = 24 h, T = (20 ± 4) °C; bars represents the fractionation of the Cr(VI) species for C_Cr(VI)_ = 104 mg L^−1^; error bars denote standard deviations from three replicates).

Figure 3 presents the effect of pH on Cr(VI) adsorption onto the studied biochar samples. In each case, the maximum Cr(VI) adsorption was observed in slightly acidic conditions (ZB_CO_2_ for pH_eq_ = 2.0, ZB_H_3_PO_4_ for pH_eq_ = 2.0, and ZB_CO_2__red for pH_eq_ = 2.7), for which the HCrO_4_^−^ anions were the predominant form of Cr(VI).

At pH values < 2, the share of the undissociated H_2_CrO_4_ is significant. Due to the lack of a negative charge, it is not electrostatically attracted to the adsorbent surface, which results in a substantial decrease in adsorption [48,49,50]. Acidic conditions around pH = 2, in which HCrO_4_^−^ anions electrostatically attracted to the surface dominate, favor surface reduction reactions in Cr(VI) to Cr(III) [51,52,53]. Therefore, adsorption at a pH of around 2 occurs due to a complex process; this includes the binding of HCrO_4_^−^ to positively charged biochar surface groups and the reduction of Cr(VI) to Cr(III) by electron donors present on the surface of the biochar. The reduced Cr(III) may be complexed with adjacent functional groups or released into water as a result of repulsion with the positively charged surface. The release of Cr(III) ions into water probably occurs in the case of ZB_CO_2_ and ZB_CO_2__red at a pH of about 1; under these conditions, the surface of these materials has a large positive charge and repulses Cr(III) cations. In the case of ZB_H_3_PO_4_, whose pH_pzc_ is lower than for ZB_CO_2_ and ZB_CO_2__red, the surface is also positively charged at pH = 1, but this charge is smaller than for the other studied materials. Hence, Cr(III) repulsion is weaker, and the adsorption value is not drastically lower than in pH = 2. With an increase in pH above 2, the adsorption of chromates decreases due to the decrease in the positive charge of the adsorbents and the decrease in the electrostatic attraction of the adsorbed anions. Thus, the main factor governing adsorption in this pH range could be the electrostatic attraction of the negatively charged HCrO_4_^−^ species by the positively charged carbonaceous surface. The adsorption mechanism, including the reduction of Cr(VI) to Cr(III), is confirmed by the analyses performed using the XPS technique described in paragraph 2.4. They indicate the reduction of Cr(VI) to Cr(III) and, therefore, the significant participation of redox processes.

Further Cr(VI) adsorption experiments were conducted at pH 2.0–2.7, where the maximum Cr(VI) adsorption values were obtained for the studied biochar materials.

**Figure 4:**
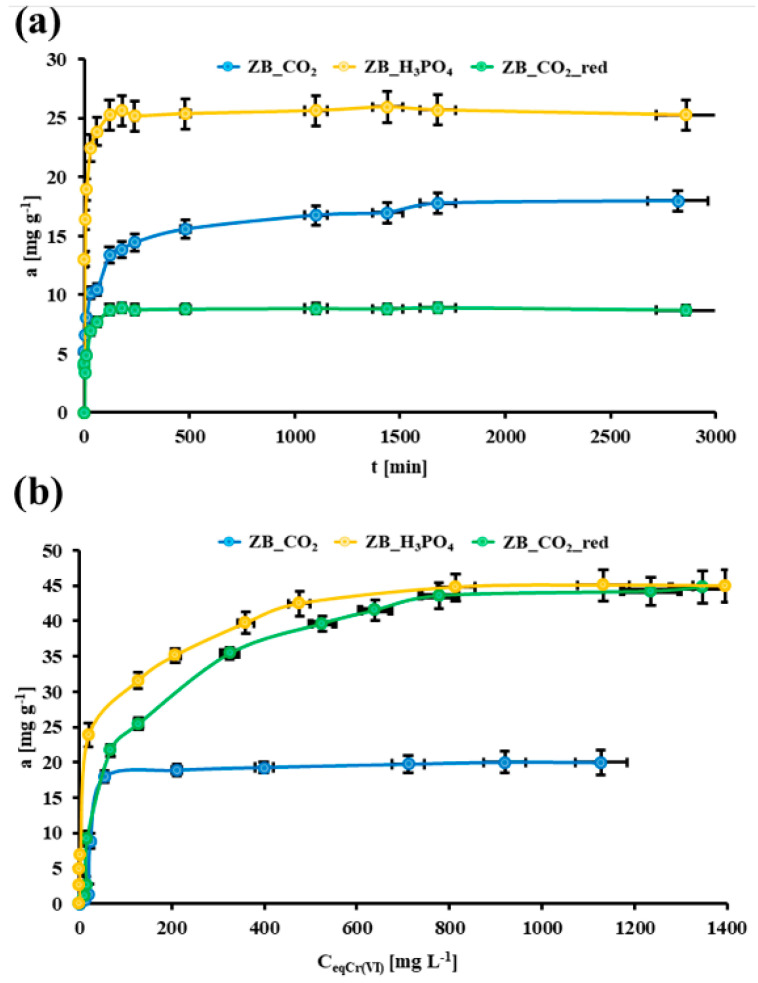
(**a**) Cr(VI) adsorption kinetics curves for the studied biochar materials (m = 20 mg, V = 5 mL, pH_eq(ZB_CO2, ZB_H3PO4)_ = 2.0, pH_eq(ZB_CO2_red)_ = 2.5, C_inCr(VI)_ZB_CO2_ = 129 mg L^−1^, C_inCr(VI)_ZB_CO2_red_ = 85 mg L^−1^, C_inCr(VI)_ZB_H3PO4_ = 119 mg L^−1^, T = (20 ± 4) °C) (**b**) The Cr(VI) adsorption isotherms for the studied biochar materials (m = 20 mg, V = 5 mL, pH_eq(ZB_CO2, ZB_H3PO4)_ = 2.0, pH_eq(ZB_CO2_red)_ = 2.5, t_eq(ZB_CO2)_ = 1100 min, t_eq(ZB_CO2_red, ZB_H3PO4)_ = 120 min, T = (20 ± 4) °C); error bars denote standard deviations from three replicates.

Figure 4a presents the Cr(VI) adsorption vs. time for all studied biochar samples. The adsorption equilibrium state was established after 1100 min for ZB_CO_2_ and 120 min for ZB_CO_2__red and ZB_H_3_PO_4_. The relatively fast adsorption process for each biochar could be related to the presence of mesopores in the biochar’s porous structure. In each case, the two-step adsorption process was observed: a fast step, probably related to Cr(VI) diffusion through mesopores, and a slow step, perhaps corresponding with the adsorption of Cr(VI) ions onto the biochar surface.

The experimental kinetics data were fitted to four theoretical kinetics models (pseudo-first-order (Equation (1)), pseudo-second-order (Equation (2)), Elovich (Equation (3)), and intraparticle diffusion (Equation (4)) [29,54] to come close to the Cr(VI) adsorption mechanism on the studied biochar materials. The following linear equations describe the abovementioned models [29,54]:

ln(q_eq_ − q_t_) = lnq_eq_ − k_1_t (1)

(2) $$\frac{1}{q_{t}}=\frac{1}{q_{eq}}+\frac{1}{q_{eq}^{2}k_{2}t}$$

q_t_ = 1/β ln(αβ) + 1/β lnt (3)

q_t_ = k_id_t^1/2^ + C (4)

where q_eq_ and q_t_ are the equilibrium Cr(VI) adsorption and Cr(VI) adsorption at time t, respectively [mg g^−1^], t is the adsorption time [min], k_1_ is the kinetics rate constant for the pseudo-first-order model [min^−1^], k_2_ is the kinetics rate constant for the pseudo-second-order model [g mg^−1^ min^−1^], α is the initial adsorption rate [mg g^−1^ min^−1^], β is the constant connected with the chemisorption activation energy and the extent of surface coverage [g mg^−1^], k_id_ is the intraparticle diffusion rate constant [mg g^−1^ min^−1/2^] and C is related to the thickness of the boundary layer; the more significant the C value was, the greater the boundary layer effect was [29,54]. Table 3 presents the results of the experimental kinetics data fitting with theoretical kinetics models. For all the biochar samples studied, the Elovich model best describes the Cr(VI) adsorption kinetics on their surface (the highest R^2^ values). In the Elovich model, chemisorption was the main factor influencing the adsorption kinetics. Moreover, the highest α value was observed for the ZB_H_3_PO_4_ biochar (8399 mg g^−1^ min^−1/2^), which means that this biochar had the most dynamic first kinetics step during the Cr(VI) adsorption process. The second parameter in the Elovich model (β) was relatively low for all studied biochar samples, which suggested the low activation energy of Cr(VI) adsorption on their surface, and indicated the high affinity of the material’s surface to the Cr(VI) ions. Only in the case of the ZB_CO_2_ material did the intraparticle diffusion have the highest contribution to the Cr(VI) adsorption kinetics (R^2^ = 0.710). For other materials, this process was not the rate-limiting step.

In Figure 4b, the Cr(VI) adsorption isotherms for all studied biochar samples are presented. The highest Cr(VI) adsorption capacity was obtained for the ZB_H_3_PO_4_ biochar (45.0 mg g^−1^) and the lowest was obtained for the ZB_CO_2_ biochar (20.0 mg g^−1^). Additionally, the paraxial character of the first part of the isotherm was expressed in the case of the ZB_H_3_PO_4_ biochar. This material could be ideal for efficient Cr(VI) removal from wastewater, and because of that, it was selected for further Cr(VI) removal studies.

Table 4 presents the fitting data of the Cr(VI) experimental adsorption isotherms with the theoretical models (Langmuir and Freundlich). The linear forms of the Langmuir (Equation (5)) and Freundlich (Equation (6)) adsorption isotherms are as follows [29,55]:

(5) $$\frac{1}{q_{eq}}=\frac{1}{q_{m}}+\frac{1}{C_{eq}q_{m}k_{L}}$$

(6) $$\ln\left(q_{eq}\right)=\ln\left(k_{F}\right)+\frac{1}{n_{F}}\ln\left(C_{eq}\right)$$

where q_m_ is the monolayer capacity [mg g^−1^], k_L_ is the Langmuir equilibrium constant [L mg^−1^], k_F_ is the Freundlich equilibrium constant [mg^1−nF^ L^nF^ g^−1^] and n_F_ is the Freundlich constant [a. u.] [29]. For all studied adsorption systems, the Freundlich model was better fitted to the experimental Cr(VI) adsorption isotherm data (ZB_CO_2_: R^2^ = 0.616; ZB_CO_2__red: R^2^ = 0.840; ZB_H_3_PO_4_: R^2^ = 0.965) than the Langmuir model. Moreover, for all biochar materials, the n_F_ > 1. This meant that the adsorption process is favorable. As a larger *n*_*F*_ value indicates higher adsorption affinity it can be concluded that ZB_H_3_PO_4_, for which *n*_*F*_ = 2.84, has the highest adsorption affinity towards Cr(VI) ions [56]. The surface of ZB_H_3_PO_4_ biochar samples was the most energetically heterogenous (1/n_F_ = 0.35) [57].

### 2.3. Cr(VI) Removal from the Brick-Originated Wastewater

**Figure 5:**
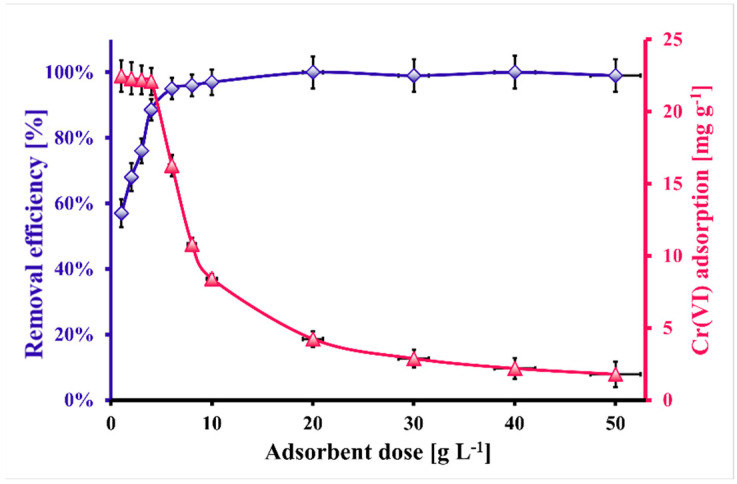
The effect of ZB_H_3_PO_4_ biochar dosage on Cr(VI) removal efficiency and Cr(VI) adsorption (pH_eq_ = 2.0, C_inCr(VI)_ = 104.7 mg·L^−1^, t_eq_ = 120 min, V = 5 mL, T = (20 ± 4) °C); error bars denote standard deviations from three replicates.

Figure 5 presents the Cr(VI) removal efficiency and adsorption capacity of ZB_H_3_PO_4_ versus biochar dosage for the adsorption systems containing extract from the magnesia–chrome brick. It was experimentally stated that the brick-originated wastewater contained mainly Ca(II) and Mg(II) ions, without iron ions, which had a negligible impact on the Cr(VI) adsorption process [22]. The higher the adsorbent dose, the lower the Cr(VI) adsorption (from 24.0 mg g^−1^ down to 1.8 mg g^−1^). The opposite situation was observed for the Cr(VI) removal efficiency, which increased from ca. 58% to 100% in the biochar dosage range 1–20 g L^−1^ and remained constant. The initial (for small dosage) decrease in Cr(VI) adsorption with the increasing dosage of biochar could be related to the cohesive interaction of the biochar particles (aggregation/agglomeration) and the simultaneous decrease in the effective surface area per unit weight (g) of the adsorbent [58]. On the contrary, the initial increase in Cr(VI) removal from the wastewater originating from spent brick could be related to the introduction of increasing amounts of biochar with the surface available for Cr(VI) ions up to a point, where the higher biochar dosage did not affect the Cr(VI) removal efficiency. The optimal biochar dosage (4.0 g L^−1^) is related to the maximum possible Cr(VI) removal efficiency for the highest Cr(VI) adsorption. The relatively low optimal dosage of the used biochar can be an advantage from an economic, environmental, and engineering point of view. Applying a low amount of biochar could lead to lower production costs, less waste generation, and the more straightforward construction of Cr(VI) removal systems (lower flow resistance). However, it should be noted that the optimal dose of sorbent will depend on the adsorbate concentration in the aqueous phase and the other chemical compounds accompanying it in the aqueous sample.

### 2.4. Cr(VI) Adsorption Mechanism

**Figure 6:**
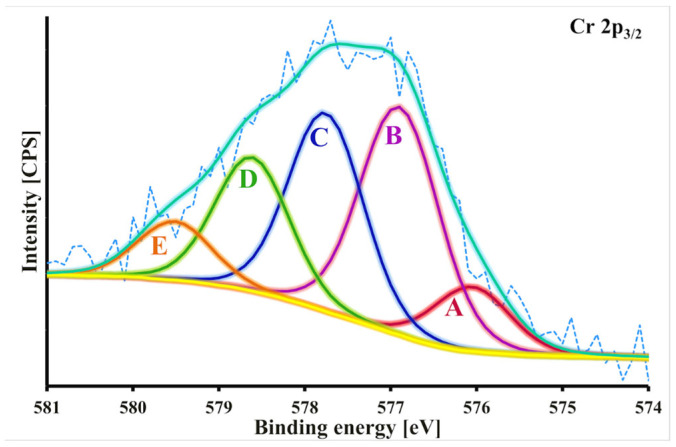
The deconvoluted Cr 2p_3/2_ spectrum for Cr-loaded ZB_H_3_PO_4_ biochar (A, B, C, D and E represents the resulted deconvoluted peaks).

**Figure 7:**
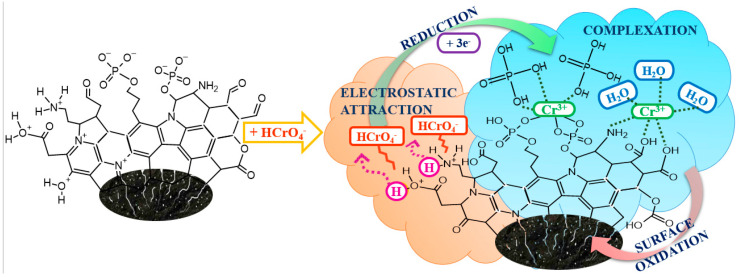
The proposed mechanism of Cr(VI) adsorption onto ZB_H_3_PO_4_ biochar.

The XPS spectra for the pristine and chromium-loaded ZB_H_3_PO_4_ biochar were recorded to study the mechanism of Cr(VI) ions adsorption from an aqueous solution onto the ZB_H_3_PO_4_ biochar in detail. The XPS results obtained after the deconvolution of the C 1s, O 1s, N 1s, P 2p, and Cr 2p core energy levels for the studied samples are presented in Table S1. Cr(VI) adsorption onto the ZB_H_3_PO_4_ biochar resulted in a decrease in the content of aliphatic carbon (from 31.7% to 7.5%) and an increase in the content of carbonyl (from 1.2% to 4.2%) and carboxyl (from 1.1% to 4.8%) groups. The O 1s data confirmed these observations. Additionally, the presence of defective carbon structures, carbonates, protonated phosphorus groups, and Cr(III) in the form of Cr_2_O_3_ (Figure 6) on the surface of chromium-loaded ZB_H_3_PO_4_ biochar was noted. During the adsorption of Cr(VI), the amine, pyridine, and quaternary nitrogen content decreased (from 82.3% to 72.5%). Simultaneously, the content of protonated imine increased from 17.7 to 22.7%, and N–O groups also appeared. Based on these observations, it could be suggested that the surface of ZB_H_3_PO_4_ biochar was oxidized during Cr(VI) adsorption. Cr(VI) present in the solution as HCrO_4_^−^ was electrostatically attracted to the positively charged surface of ZB_H_3_PO_4_. The dependence of adsorption on pH evidences the strong influence of electrostatic interactions on adsorption. The surface charge of the sorbent determines adsorption. Therefore, electrostatic interactions play a key role in the adsorption process. After attracting Cr(VI) to the positively charged surface, the HCrO_4_^−^ ions present in the aqueous solution could be reduced to Cr(III) by the sorbent, with the simultaneous oxidation of its surface. In the XPS spectrum of Cr-loaded ZB_CO_2_, in the region of the Cr 2 p 3/2 binding energy level, three peaks with binding energies of 576.1, 576.9 and 577.7 eV corresponding to different forms of Cr(III) are registered, indicating that more than 70% of the chromium present on the carbon surface is in its reduced form [59,60]. Both the minority Cr(VI) and dominant Cr(III) ions could be complexed by the surface carboxyl, amine, and phosphorus groups (Figure 7) [38,39,40,41,42,43,44,45,46,58]. Adsorption is a complex process that includes phenomena such as electrostatic interaction, reduction, and complexation.

## 3. Materials and Methods

### 3.1. Raw Material and Reagents

The raw slumgum was obtained from the professional “Miodek” Apiary Farm in Lublin (Poland) [61], cleaned of metal residues using a 1 T magnet to eliminate secondary water contamination, and denoted as ZB.

The chemicals used in this work were as follows: nitric acid (65 wt. %, Suprapur, Chempur, Piekary Śląskie, Poland), potassium dichromate (≥90 wt. %, Merck, Darmstadt, Germany), sodium hydroxide (≥90 wt. %, Stanlab, Lublin, Poland), orthophosphoric acid (85 wt. %, pure p.a, Erba Lachema, Brno, Czech Republic), hydrochloric acid (35 wt. %, pure p.a, Polish Chemical Reagents, Gliwice, Poland), iron(III) nitrate nonahydrate (≥90 wt. %, Merck, Darmstadt, Germany), Cr(VI) standard solution (1000 mg L^−1^, Merck, Darmstadt, Germany), carbon dioxide (99,995%, Air Liquide Poland, Kraków, Poland), argon and nitrogen (99%, Air Liquide Poland, Kraków, Poland). Distilled Milli-Q water from Millipore (Merck, Darmstadt, Germany) was used throughout all studies.

The spent magnesia–chrome refractory bricks used in this study were obtained from industrial cement work waste (south-eastern Poland) [22].

### 3.2. Preparation of Biochar Materials

Preliminary preparation of the raw material: The raw ZB material was manually stripped of wires and dried at 120 °C overnight in the laboratory oven. The prepared ZB material was chemically modified as follows.

Activation-modification by CO_2_: Firstly, 20 g of the preliminary prepared ZB raw material was heated at 350 °C for 1 h under a CO_2_ atmosphere (100 mL min^−1^) in a quartz tubular furnace. The obtained material was ground in an agate ball mill in the next step. The milled material was pyrolyzed in the tubular quartz furnace at 800 °C [23] for 5 h under a CO_2_ atmosphere (100 mL min^−1^). The obtained biochar was washed several times with 1 mol L^−1^ HCl and thoroughly washed with double-distilled water to a pH close to 7. Finally, the activated biochar was dried overnight in the oven at 120 °C. The CO_2_-activated biochar from the slumgum was denoted as ZB_CO_2_.

High-temperature reduction: First, 3 g of ZB_CO_2_ was placed in the quartz tube and pyrolyzed under the fluidized Ar flow at 1000 °C for 30 min. The obtained biochar was denoted as ZB_CO_2__red.

Activation-modification by H_3_PO_4_: First, 20 g of the ZB material was immersed in 74.6 mL of 60 wt. % H_3_PO_4_ for 3 h; the obtained mixture was dried in the muffle furnace at 220 °C overnight. The top layer containing waxes was separated from the received product and the black, mushy residue was pyrolyzed in the quartz tubular furnace under a nitrogen atmosphere (1 L min^−1^) according to the following time–temperature program: 25–360 °C (10 °C min^−1^), 360–450 °C (2 °C min^−1^), 450–700 °C (10 °C min^−1^) and 700 °C for 3 h. The modified biochar was washed several times with deionized water until orthophosphates were absent in the filtrate (precipitation reaction with the Fe^3+^ ions), and it was dried at 120 °C overnight. The obtained biochar was denoted as ZB_H_3_PO_4_.

**Figure 8:**
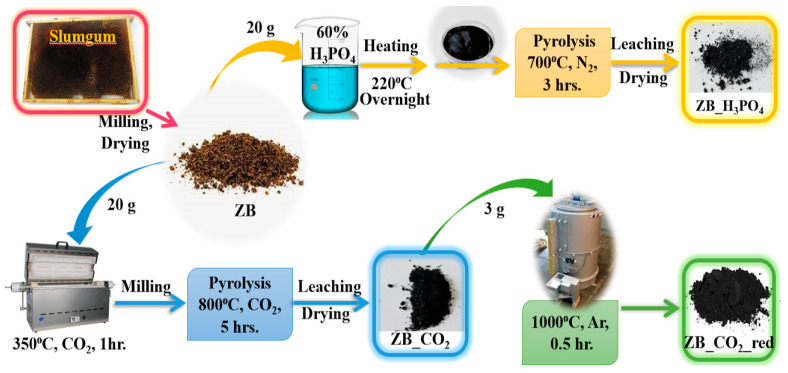
The synthesis scheme for materials originating from the slumgum waste.

The process of synthesizing the biochar materials is illustrated in Figure 8. For each modification, 3 samples were prepared and physicochemically characterized. Next, the samples regarding the exact modification method were homogenized and used for further experiments.

### 3.3. Physicochemical Characteristics

The ASAP 2420 analyzer (Micromeritics Inc., Norcross, GA, USA) was utilized to determine the nitrogen adsorption/desorption isotherms for the studied materials at −196 °C. All samples were degassed at 120 °C in a vacuum for 12 h before measurements. In each case, the adsorption branch of the nitrogen adsorption/desorption isotherm was used for the estimation of the BET surface area (S_BET_), total pore volume (V_Tot._), and BJH pore size distribution (PSD). The elemental analysis of biochar was performed using the CHN analyzer EuroEA 3000 (EuroVector, Pavia, Italy). The XPS and attenuated total reflectance (ATR) with Fourier transform infrared (FT–IR) spectroscopy techniques were applied to examine the surface functional groups in the studied biochar samples. X-ray photoelectron spectroscopy measurements were performed with a Multi-Chamber Analytical System (Prevac, Rogów, Poland) equipped with monochromatic K_α_-Al radiation (1486.6 eV) (Gammadata Scienta, Uppsala, Sweden) and an X-ray power of 450 W. All binding energies were referenced to the carbon C 1s peak at 285 eV. The ATR FT–IR spectra were recorded by the Bruker TENSOR 27 FTIR spectrophotometer equipped with a diamond crystal. For each sample, 32 scans in the 600–4000 cm^−1^ range were recorded.

A pH meter CP-401 (Elmetron, Zabrze, Poland) equipped with a glass electrode was used for pH measurements. The acid–base properties of the studied biochar materials (pH_pzc_) were examined by measuring the equilibrium pH (pH_f_) of aqueous 0.01 mol L^−1^ KCl solutions, which were obtained after the 24 h shaking of suspensions composed of 20 mg of adsorbent and 5 mL of 0.01 mol L^−1^ KCl with an initial pH (pH_i_) in the range of 2–12. The difference between the initial and equilibrium pH was calculated as follows: ∆pH = pH_i_ − pH_f_. The ∆pH was plotted as a function of pH_i_. The pH_i_ value with ∆pH equal to zero was the zero point of charge, pH_pzc_, of the material [62].

A flame atomic absorption spectrometer (F AAS) (SpectrAA 880, Varian, Australia) equipped with an air–acetylene burner was used for chromium determination in liquid phases. The working parameters were as follows: spectral bandwidth of 0.2 nm, air-to-acetylene flow ratio of 4.5, and no spectral correction. The F AAS measurements were performed using a Cr hollow cathode lamp (Varian, Australia) at a wavelength of 357.9 nm and with a 7 mA lamp current. Before measurements, the spectrometer was calibrated against a Cr(VI) standard solution.

### 3.4. Cr(VI) Batch Adsorption Studies

All Cr(VI) adsorption experiments were conducted at 25 °C. The 20 mg of adsorbent was briefly immersed in 5 mL of an aqueous Cr(VI) solution in a 25 mL Erlenmeyer flask, and the suspension was shaken at 120 rpm for 48 h (mechanical shaker equipped with thermostate Elpan 357 (Elpin Plus, Lubawa, Poland)). The exception was kinetics studies, where the contact time was 1–2860 min. The isotherm data were collected for initial Cr(VI) concentrations between 1.0 mg L^−1^ and 1.3 g L^−1^. After a defined contact time, the solid adsorbent was separated from the liquid phase by centrifugation. The initial and equilibrium Cr concentrations were determined in the liquid phase using the FAAS technique.

The amount of Cr(VI) (a [mg g^−1^]) adsorbed onto the surface of the studied material was calculated according to the following equation:

(7) $$a=\frac{\left(C_{inCr(VI)}-C_{eqCr(VI)}\right)V}{m}$$

where C_inCr(VI)_ is the initial Cr(VI) concentration in the liquid phase [mg L^−1^], C_eqCr(VI)_ is the equilibrium Cr(VI) concentration in the liquid phase [mg L^−1^], V is the volume of the liquid phase [mL] and m is the adsorbent mass [mg].

### 3.5. Removal of Cr(VI) from Brick-Originated Wastewater

The brick-originated wastewater was prepared as follows: 40.0 g of milled spent magnesia–chrome brick (particle diameter < 0.75 mm) was mixed with 200 mL of distilled water and shaken for 48 h. After filtration, the extract was collected in a conical flask. The measured chromium concentration in an aqueous extract was 104.7 mg L^−1^ Cr(VI).

In each Cr(VI) removal experiment, 5–250 mg portions of ZB_H_3_PO_4_ biochar were mixed with 5 mL of brick-originated wastewater. The pH of the wastewater was adjusted to 2.0, and the carbonaceous suspension was shaken for 48 h. Next, the solid adsorbent was separated from the liquid phase by centrifugation. The initial and equilibrium Cr concentrations were determined in the liquid phase using the FAAS technique. The Cr(VI) removal efficiency (RE [%]) (Equation (8)) was calculated as follows:

(8) $$RE\left[\%\right]=\frac{\left(C_{inCr(VI)}-C_{eqCr(VI)}\right)}{C_{inCr(VI)}}100\%$$

## 4. Conclusions

For the first time, beekeeping waste was successfully used to synthesize biochar materials with a micro-mesoporous structure. The H_3_PO_4_ treatment of the pristine material was the most effective way to obtain biochar with a high specific surface area (639 m^2^ g^−1^) and a heteroatoms-rich (N, O, P) carbonaceous surface. The highest static adsorption capacity towards Cr(VI) ions was evaluated for the biochar activated with H_3_PO_4_ (45.0 mg g^−1^) at pH = 2.0 after 120 min. The mechanism of Cr(VI) adsorption onto the H_3_PO_4_-modified biochar was complicated. It consisted of the electrostatic attraction of HCrO_4_^−^ ions to the positively charged biochar surface, the reduction of more toxic chromium species (Cr(VI)) to less toxic species (Cr(III)), and the surface complexation of Cr(III) ions [63].

The H_3_PO_4_-modified biochar was successfully applied for the efficient removal of Cr(VI) from wastewater originating from spent bricks. A high Cr(VI) removal efficiency (close to 100%) was obtained for the relatively low biochar dosage (4.0 g L^−1^). A low optimal dosage of biochar can reduce the cost of producing biochar and lead to the generation of less waste. It also allows for the simplification of the construction of Cr(VI) removal systems. Moreover, the toxicity of the wastewater containing Cr(VI) ions can be lowered by using the synthesized biochar.

## Figures and Tables

**Table 1:** Porosity parameters of the pristine slumgum and various biochar materials estimated based on the nitrogen adsorption/desorption isotherms.

Material Symbol	S_BET_ [m^2^ g^−1^]	V_Tot._ [cm^3^ g^−1^]	d_BJH,des._ [nm]
ZB	0 *	0 *	11.9 ^$^ ± 0.4 ^#^
ZB_CO_2_	254 ^$^ ± 8 ^#^	0.17 ^$^ ± 0.01 ^#^	4.4 ^$^ ± 0.1 ^#^
ZB_CO_2__red	105 ^$^ ± 4 ^#^	0.12 ^$^ ± 0.01 ^#^	5.7 ^$^ ± 0.2 ^#^
ZB_H_3_PO_4_	663 ^$^ ± 10 ^#^	0.39 ^$^ ± 0.02 ^#^	3.5 ^$^ ± 0.1 ^#^

* close to 0; ^$^—mean value from three independent replicates; ^#^—the SD value from three independent replicates.

**Table 2:** Elemental content of the studied materials.

Material	CHN			XPS				
	C [wt. %]	H [wt. %]	N [wt. %]	C [wt. %]	N [wt. %]	O [wt. %]	P [wt. %]	K [wt. %]
ZB	59.2 ^$^ ± 2.4 ^#^	10.7 ^$^ ± 0.4 ^#^	5.2 ^$^ ± 0.3 ^#^	nd	nd	nd	nd	nd
ZB_CO_2__red	68.3 ^$^ ± 1.7 ^#^	1.6 ^$^ ± 0.1 ^#^	3.9 ^$^ ± 0.2 ^#^	71.4 ^$^ ± 1.8 ^#^	2.6 ^$^ ± 0.1 ^#^	17.2 ^$^ ± 0.6 ^#^	1.8 ^$^ ± 0.1 ^#^	5.1 ^$^ ± 0.2 ^#^
ZB_CO_2_	84.7 ^$^ ± 1.2 ^#^	2.9 ^$^ ± 0.3 ^#^	3.8 ^$^ ± 0.1 ^#^	88.8 ^$^ ± 1.3 ^#^	2.1 ^$^ ± 0.2 ^#^	7.6 ^$^ ± 0.4 ^#^	1.9 ^$^ ± 0.1 ^#^	4.9 ^$^ ± 0.2 ^#^
ZB_H_3_PO_4_	52.1 ^$^ ± 2.3 ^#^	3.4 ^$^ ± 0.3 ^#^	3.9 ^$^ ± 0.1 ^#^	65.9 ^$^ ± 3.1 ^#^	3.9 ^$^ ± 0.1 ^#^	20.6 ^$^ ± 0.9 ^#^	8.1 ^$^ ± 0.3 ^#^	0 *

*—close to 0; ^$^—mean value from three independent replicates; nd—no data; ^#^—the SD value from three independent replicates.

**Table 3:** Parameters of Cr(VI) adsorption kinetics data fitting to the theoretical kinetics models, i.e., pseudo-first order (PFO) model, Elovich model and intraparticle diffusion model (IPD) for the studied biochar materials.

Material	PFO			PSO			Elovich				IPD		
	q_eq.calc._ [mg g^−1^]	k_1_ [min^−1^]	R^2^	q_eq.calc._ [mg g^−1^]	k_2_ [g mg^−1^ min^−1^]	R^2^	q_eq.exp._ [mg g^−1^]	α [mg g^−1^ min^−1^]	β [g mg^−1^]	R^2^	k_id_ [mg g^−1^ min^−1/2^]	C [mg g^−1^]	R^2^
ZB_CO_2_	8.96 ^$^ ± 0.45 ^#^	0.002 ^$&^	0.890	13.05 ^$^ ± 0.73 ^#^	0.046 ^$&^	0.705	17.9 ^$^ ± 0.9 ^#^	21.1 ^$^ ± 1.1 ^#^	0.57 ^$^ ± 0.03 ^#^	0.984	0.27 ^$^ ± 0.01 ^#^	7.26 ^$^ ± 0.36 ^#^	0.710
ZB_CO_2__red	1.40 ^$^ ± 0.07 ^#^	0.002 ^$&^	0.408	7.50 ^$^ ± 0.03 ^#^	0.108 ^$&^	0.535	8.90 ^$^ ± 0.45 ^#^	115 ^$^ ± 5 ^#^	1.32 ^$^ ± 0.07 ^#^	0.840	0.11 ^$&^	4.94 ^$^ ± 0.25 ^#^	0.454
ZB_H_3_PO_4_	3.21 ^$^ ± 0.16 ^#^	0.001 ^$&^	0.295	24.0 ^$^ ± 0.9 ^#^	0.039 ^$&^	0.820	25.9 ^$^ ± 1.3 ^#^	8399 ^$^ ± 420 ^#^	0.59 ^$^ ± 0.02 ^#^	0.848	0.26 ^$^ ± 0.01 ^#^	16.4 ^$^ ± 0.8 ^#^	0.372

^$^—mean value from three independent replicates; ^#^—the SD value from three independent replicates, ^&^—SD close to zero.

**Table 4:** The fitting data of the Cr(VI) experimental adsorption isotherms with the theoretical models (Langmuir and Freundlich) for all studied biochar materials.

Material	Langmuir				Freundlich		
	q_m,exp._ [mg g^−1^]	q_m,teoret._ [mg g^−1^]	k_L_ [L mg^−1^]	R^2^	n_F_ [a. u.]	k_F_ [mg^1−nF^ L^nF^ g^−1^]	R^2^
ZB_CO_2_	20.0 ^$^ ± 1.0 ^#^	80.0 ^$^ ± 4.0 ^#^	0.001 ^&^	0.562	1.37 ^$^ ± 0.02 ^#^	0.58 ^$^ ± 0.08 ^#^	0.616
ZB_CO_2__red	44.7 ^$^ ± 1.2 ^#^	103.5 ^$^ ± 2.4 ^#^	0.002 ^&^	0.833	1.48 ^$^ ± 0.03 ^#^	0.29 ^$^ ± 0.04 ^#^	0.840
ZB_H_3_PO_4_	45.0 ^$^ ± 2.3 ^#^	33.0 ^$^ ± 1.7 ^#^	0.217 ^$^ ± 0.010 ^#^	0.909	2.84 ^$^ ± 0.02 ^#^	5.25 ^$^ ± 0.32 ^#^	0.965

^$^—mean value from three independent replicates; ^#^—the SD value from three independent replicates, ^&^—SD close to zero.

## Data Availability

The raw data supporting the conclusions of this article will be made available by the authors on request.

## Supplementary Materials

The following supporting information can be downloaded at: https://www.mdpi.com/article/10.3390/molecules30112421/s1, Figure S1: Nitrogen adsorption/desorption isotherm for the ZB material; Figure S2: Pore size distribution for the ZB material; Figure S3: FT-IR spectra of studied biochars; Figure S4: Fitting of the Cr(VI) adsorption kinetics for studied materials to the pseudo-first-order and pseudo-second-order models; Figure S5: Fitting of the Cr(VI) adsorption kinetics for studied materials to the Elovich and Intraparticle diffusion (IPD) models; Figure S6: Fitting of the Cr(VI) adsorption isotherms for studied materials to the Langmuir model; Figure S7: Fitting of the Cr(VI) adsorption isotherms for studied materials to the Freundlich model; Table S1: XPS deconvolution data for ZB_H3PO4 biochar before and after Cr(VI) adsorption.
