# Supplementary material for: Removal of Hexavalent Chromium from Wastewater Originating from Spent Bricks by Modified Biochars Derived from Honeybee Biomass
Source: Molecules. 2025 May 31;30(11):2421. doi: 10.3390/molecules30112421 (PMC12155991; doi:10.3390/molecules30112421)
Supplement: Supplementary file 1 [file molecules-30-02421-s001.zip › molecules-3641774-supplementary.pdf]

REMOVAL OF HEXAVALENT CHROMIUM FROM SPENT BRICKS-ORIGINATED WASTEWATER BY MODIFIED BIOCHARS DERIVED FROM HONEYBEE BIOMASS

Authors: Rafał Olchowski[b], Kinga Morlo[a], Joanna Dobrzyńska[a], Ryszard Dobrowolski[a,*]

[a] Department of Analytical Chemistry, Institute of Chemical Sciences, Faculty of Chemistry, Maria Curie-Sklodowska University, M. C. Sklodowska Sq. 3, 20-031 Lublin, Poland

kinga.morlo@mail.umcs.pl

joanna.dobrzynska@mail.umcs.pl

[b] Department of Pharmacology, Toxicology and Environmental Protection, Faculty of Veterinary Medicine, University of Life Sciences, Akademicka St. 12, 20-950 Lublin, Poland

rafal.olchowski@up.lublin.pl

[*] **Correspondence:** ryszard.dobrowolski@mail.umcs.pl

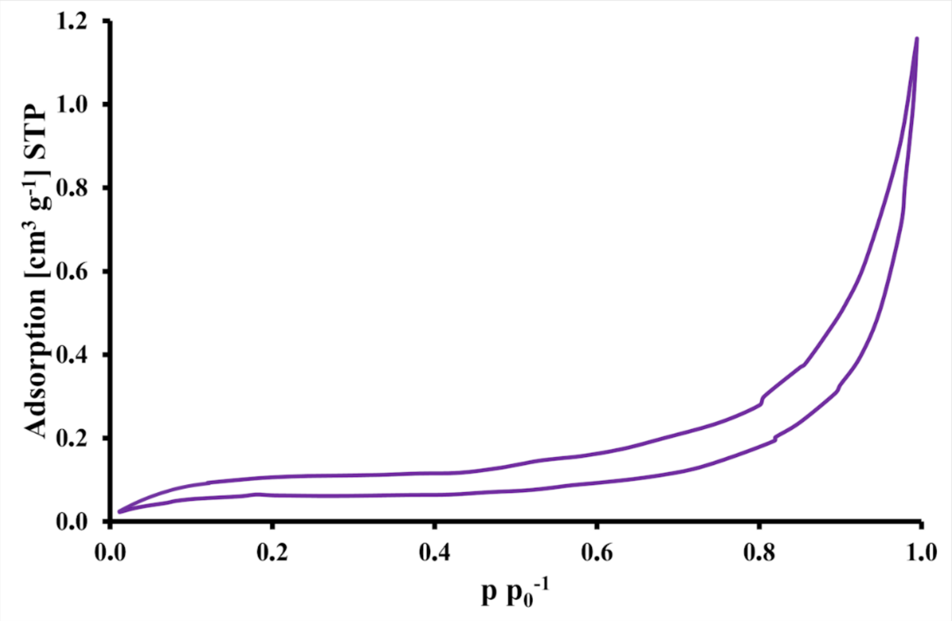

Fig. S1 Nitrogen adsorption/desorption isotherm for the ZB material

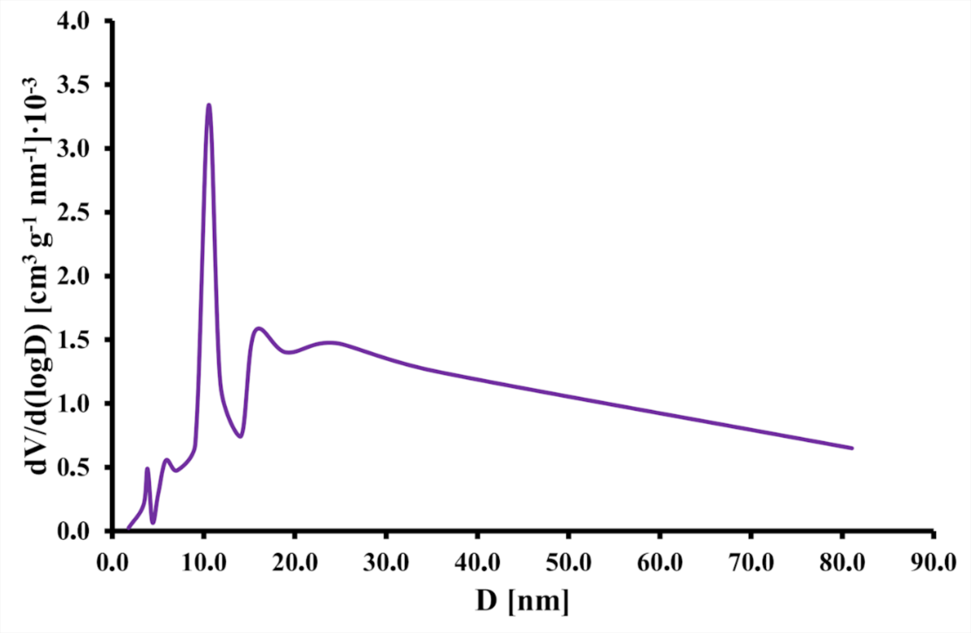

Fig. S2 Pore size distribution for the ZB material

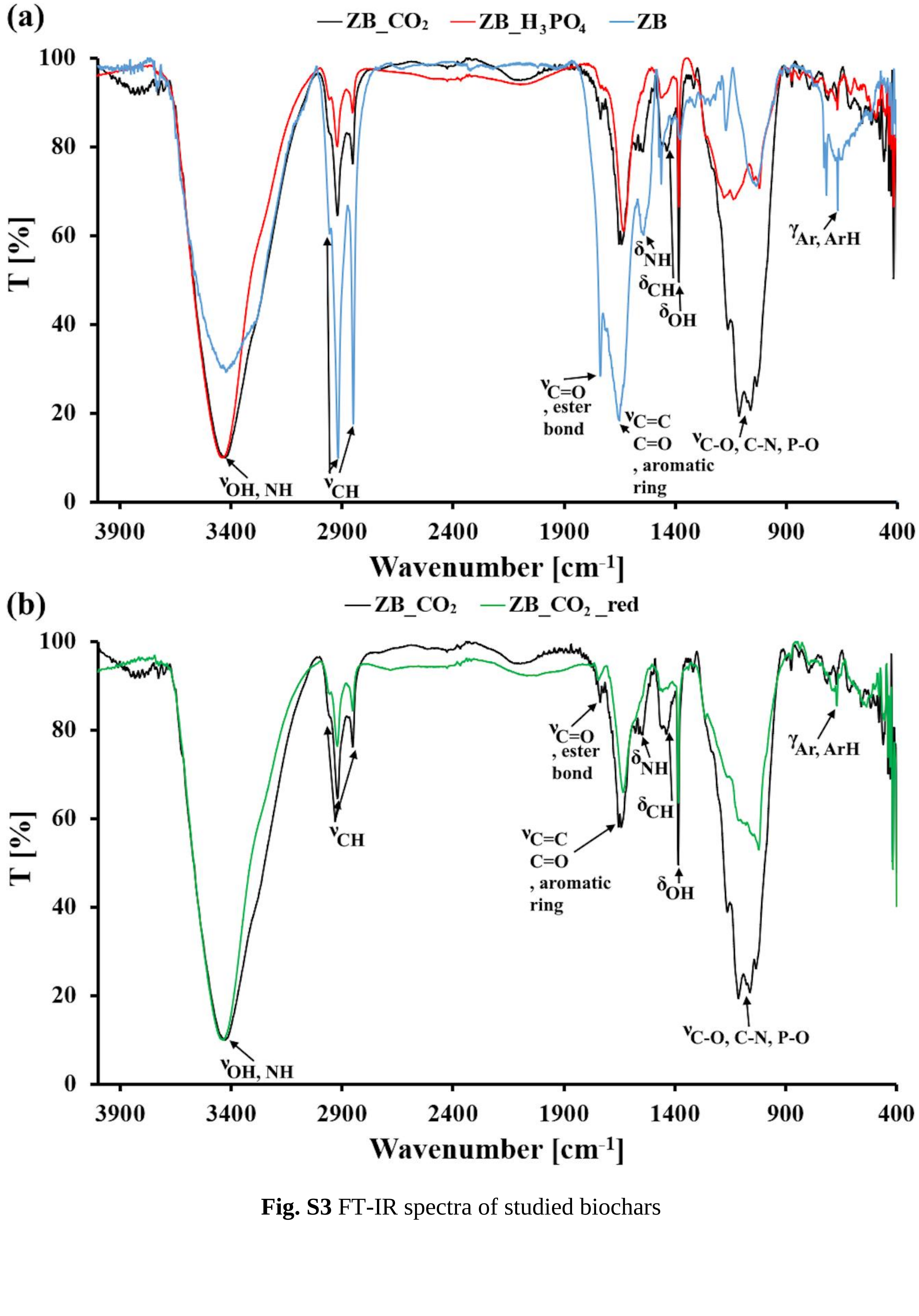

Fig. S3 FT-IR spectra of studied biochars

Tab. S1 XPS deconvolution data for ZB_H_3PO_4 biochar before and after Cr(VI) adsorption

**Binding energy [eV]**	**Functional group**	**Contribution [%]**	
		**before Cr(VI) adsorption**	**after Cr(VI) adsorption**
C 1s			
284.0	defective carbon structures	-	7.1
284.4	C=C sp^2	54.9	64.8
285.0-285.6	C-H, C-C sp^3	31.7	7.5
286.2-287.0	C-O, C-N	11.1	11.6
287.9	C=O (carbonyl)	1.2	4.2
288.9	O=C-O (carboxyl)	1.1	4.8
O 1s			
530.4	O^{2-} (metal oxides)	-	5.9
530.9-531.0	O=C, <u>O</u>=C-O, P-O	25.8	29.4
531.9	CO_3^{2-}	-	23.8
532.5-532.8	HO-C, P=O	45.3	31.5
533.5-534.1	O=C-<u>O</u>, P-OH	20.7	9.4
534.8	O_2, H_2O	8.2	-
N 1s			
398.4-399.8	amine, amide, pyrrolic, pyridine, nitrile	26.8	72.5
400.9-401.1	quaternary nitrogen	63.2	22.7
403.0-403.6	N-O	10.0	4.8
P $2p_{3/2}$			
133.0	HPO_4^{2-}	-	88.1
133.8	PO_4^{3-}	100.0	-
135.2	H_3PO_4	-	11.9
Cr $2p_{3/2}$			
576.1-579.5	Cr_2O_3	-	100

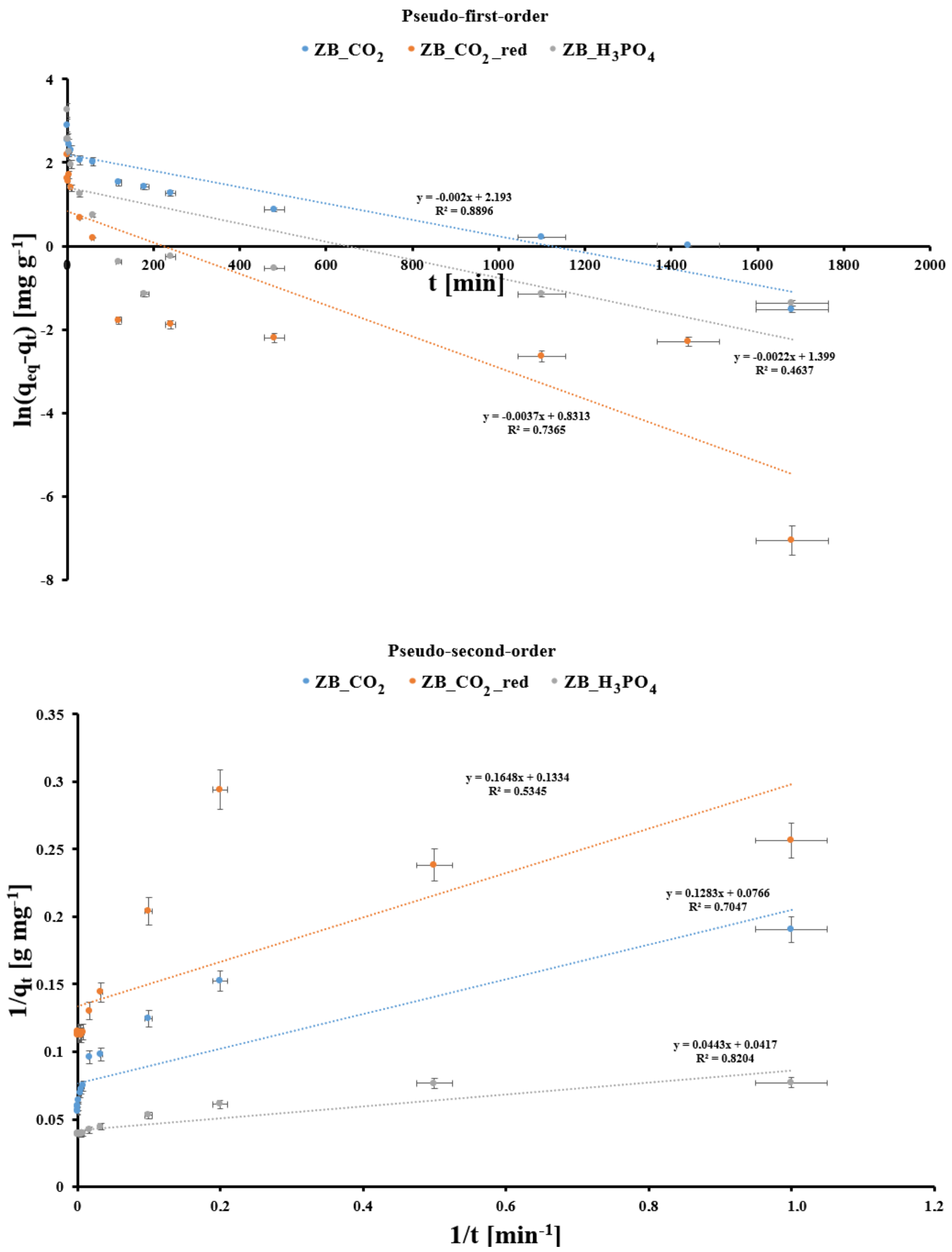

Fig. S4 Fitting of the Cr(VI) adsorption kinetics for studied materials to the pseudo-first-order and pseudo-second-order models.

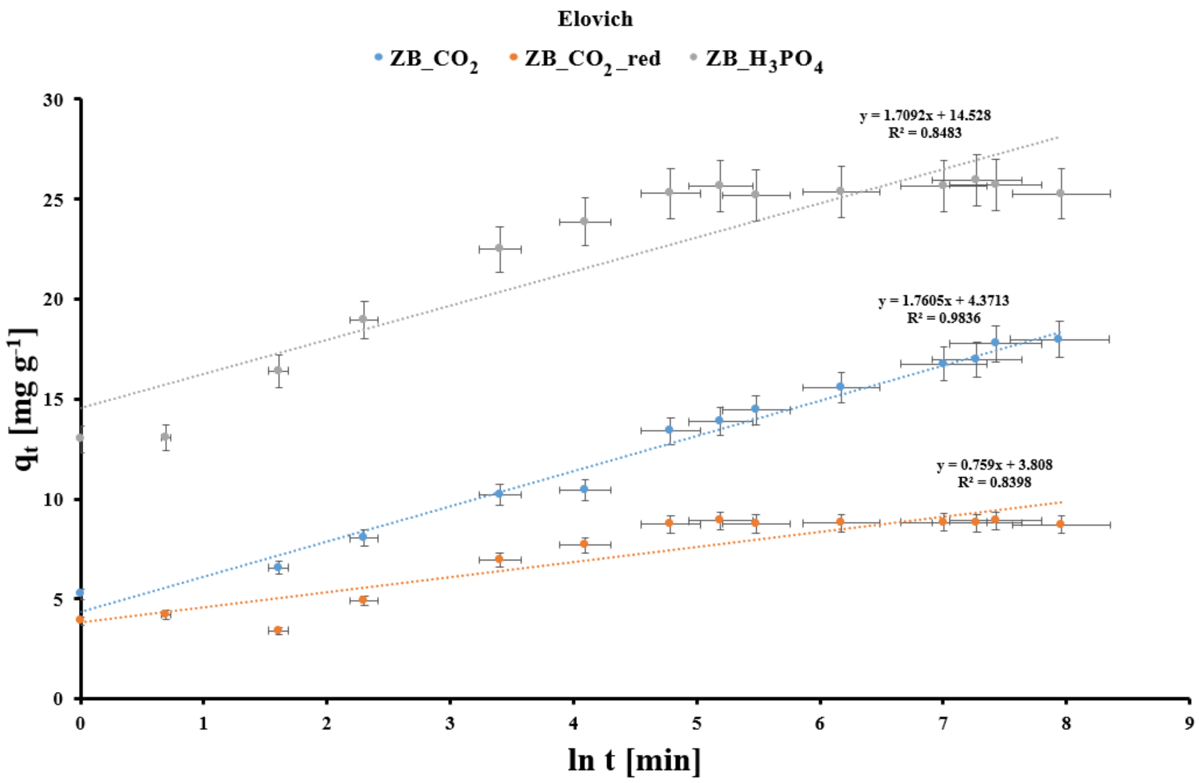

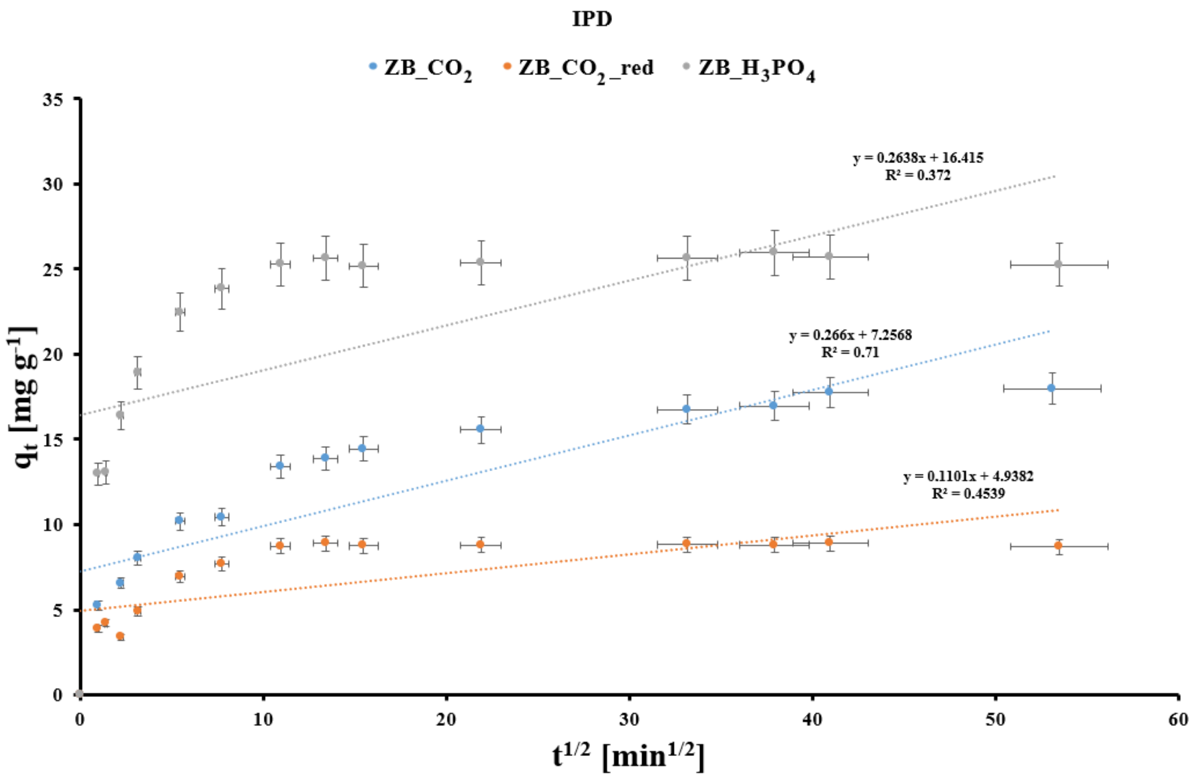

Fig. S5 Fitting of the Cr(VI) adsorption kinetics for studied materials to the Elovich and Intraparticle diffusion (IPD) models.

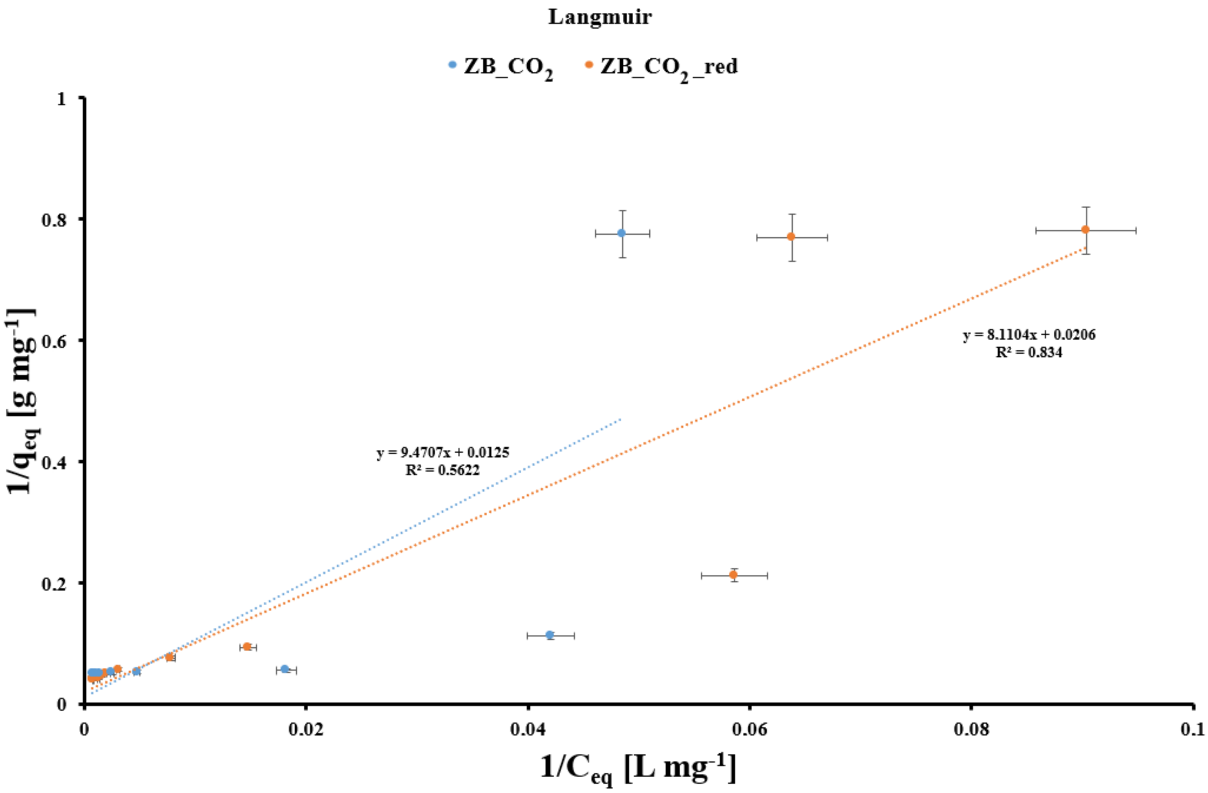

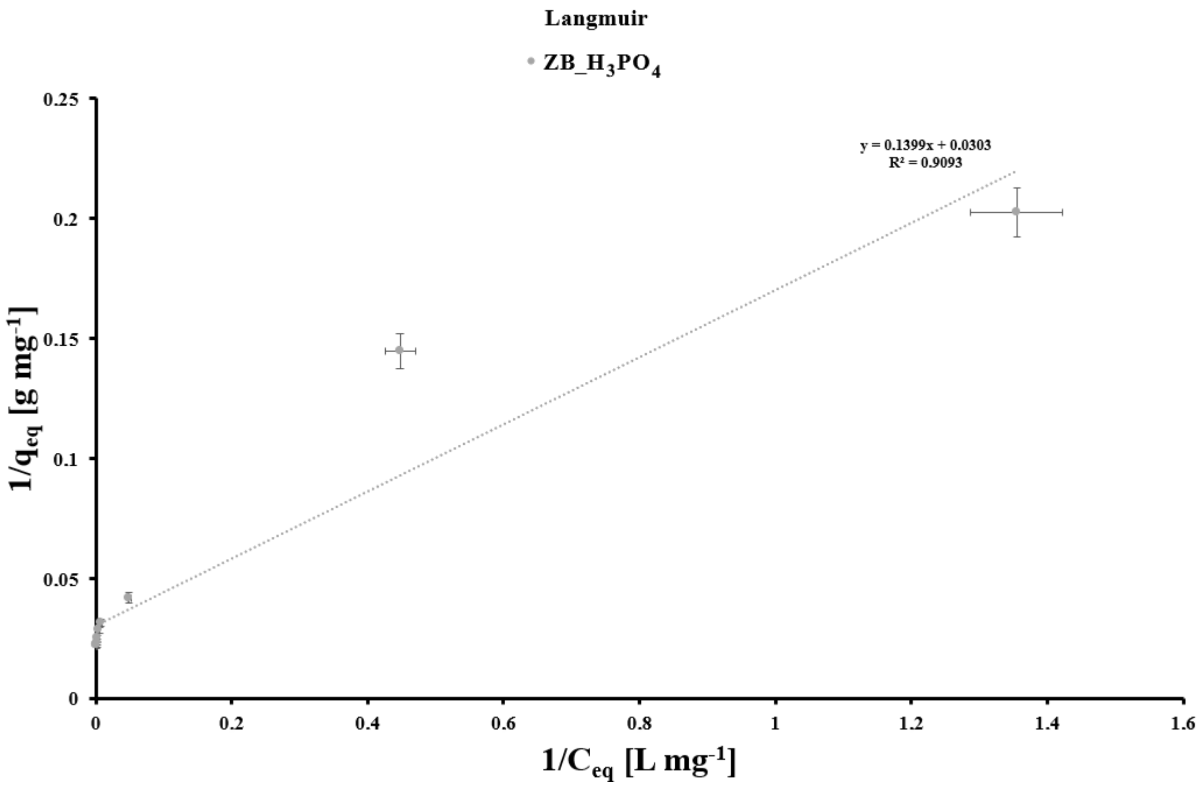

Fig. S6 Fitting of the Cr(VI) adsorption isotherms for studied materials to the Langmuir model.

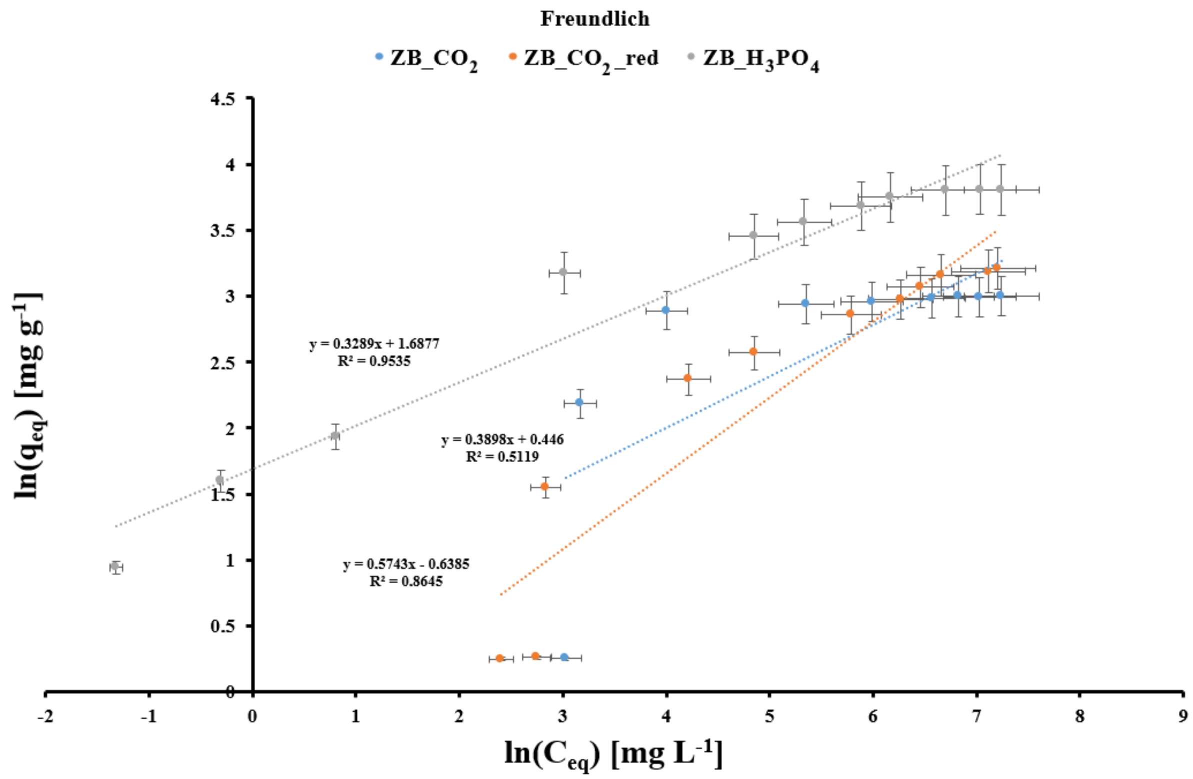

Fig. S7 Fitting of the Cr(VI) adsorption isotherms for studied materials to the Freundlich model.
